# Combination of HDAC and FYN inhibitors in synovial sarcoma treatment

**DOI:** 10.3389/fcell.2024.1422452

**Published:** 2024-07-09

**Authors:** Kyra Parker, Yanfeng Zhang, Gavin Anchondo, Ashlyn Smith, Sergio Guerrero Pacheco, Tadashi Kondo, Le Su

**Affiliations:** ^1^ Department of Biology, Jacksonville State University, Jacksonville, AL, United States; ^2^ Department of Genetics, The University of Alabama at Birmingham, Birmingham, AL, United States; ^3^ National Cancer Center, Tokyo, Japan

**Keywords:** synovial sarcoma, SS18-SSX, fyn, histone deacetylase inhibitor (HDACi), drug synergism

## Abstract

The SS18-SSX fusion protein is an oncogenic driver in synovial sarcoma. At the molecular level, SS18-SSX functions as both an activator and a repressor to coordinate transcription of different genes responsible for tumorigenesis. Here, we identify the proto-oncogene *FYN* as a new SS18-SSX target gene and examine its relation to synovial sarcoma therapy. FYN is a tyrosine kinase that promotes cancer growth, metastasis and therapeutic resistance, but SS18-SSX appears to negatively regulate *FYN* expression in synovial sarcoma cells. Using both genetic and histone deacetylase inhibitor (HDACi)-based pharmacologic approaches, we show that suppression of SS18-SSX leads to *FYN* reactivation. In support of this notion, we find that blockade of FYN activity synergistically enhances HDACi action to reduce synovial sarcoma cell proliferation and migration. Our results support a role for FYN in attenuation of anti-cancer activity upon inhibition of SS18-SSX function and demonstrate the feasibility of targeting FYN to improve the effectiveness of HDACi treatment against synovial sarcoma.

## Introduction

Synovial sarcoma is a deadly type of cancer that mostly affects children and young adults. This disease is rare in that it affects an average of one to three people in one million individuals (Rajwanshi et al., 2009; Sultan et al., 2009). Synovial sarcoma specifically targets areas of the body where the soft tissues of joints and skeletal muscle are present. The most common places that the tumors can be found are in the ankles or knees; however, metastases occur in approximately 50% of synovial sarcoma patients and have been documented to grow at distinct sites, such as the brain, liver, lung and lymph nodes (Krieg et al., 2011; Vlenterie et al., 2016). Although pain manifests around the affected areas, synovial sarcoma can be often misdiagnosed as arthritis or bursitis since the pain envelopes around the joints with no obvious changes of surrounding tissue unless checked with x-rays (Li et al., 2024). The standard treatment for synovial sarcoma, similar to other cancers, is surgery in conjunction with radiation and/or chemotherapy (Blay et al., 2023). However, patients with synovial sarcoma do not gain significant benefits, as the tumors commonly reoccur in primary lesions or metastasize to different organs.

Most, if not all, of patients that contract synovial sarcoma carry a unique gene mutation called SS18-SSX, which serves as a diagnostic biomarker in the clinic. This mutation is created by the fusion of two separate genes, namely, SS18 and SSX, through chromosomal translocation (Crew et al., 1995). The resulting SS18-SSX fusion protein retains most of the SS18 sequence, except for a C-terminal exchange with 78 amino acids from SSX (Ladanyi, 2001). Interestingly, wild-type SS18 protein is a transcriptional coactivator, while SSX acts as a mediator of repression (Brett et al., 1997; Thaete et al., 1999; dos Santos et al., 2001). After being formed, SS18-SSX behaves as a transcriptional regulator capable of both promoting and inhibiting gene expression (Nielsen et al., 2015). For example, through cooperation with the SWItch/Sucrose Non-Fermentable (SWI/SNF) chromatin remodeling complex, SS18-SSX can perturb the Polycomb repressive complex target regions leading to aberrant gene activation (Kadoch and Crabtree, 2013; Banito et al., 2018; McBride et al., 2018). Conversely, SS18-SSX can also recruit the Groucho/Polycomb repressive complex to specific gene sites required for transcriptional repression (Su et al., 2012; Cironi et al., 2016). In parallel with mechanistic studies, transgenic mouse experiments have validated the SS18-SSX fusion as a putative oncogenic driver in synovial sarcoma (Haldar et al., 2007; Haldar et al., 2009; Jones et al., 2016; Benabdallah et al., 2023). Despite the therapeutic value of SS18-SSX, its complex roles in transcriptional regulation makes synovial sarcoma very difficult to cure.

Recent evidence suggests that targeting of histone deacetylase (HDAC) family members, and of HDAC2 in particular, facilitates SS18-SSX degradation and inhibits its oncogenic activity in synovial sarcoma cells (Patel et al., 2019). While several HDAC inhibitors are currently available for synovial sarcoma trials, they have yet to be proven the most effective way of treating this deadly disease. Previous clinical studies indicated that HDAC inhibition alone is not enough to fully block synovial sarcoma growth and metastasis (Cassier et al., 2013; Chu et al., 2015). Our work has identified the proto-oncogene FYN as a direct target of SS18-SSX and shown that suppression of SS18-SSX by a potent HDAC inhibitor (FK228) increases FYN expression levels, which may in turn reduces the treatment response of synovial sarcoma cells. Moreover, we have explored the therapeutic relevance of the FYN inhibitor PP2, which synergistically interacts with FK228 and improves its efficacy in inhibiting synovial sarcoma cell proliferation and migration.

## Methods

Cell culture, chemicals and detailed procedures are described in the Supplementary Material. FLAG immunoprecipitation was performed using the M2 antibody (Sigma), as described before (Patel et al., 2019). Chromatin immunoprecipitation (ChIP) was performed using the Active Motif ChIP-IT Express kit, and ChIP DNA was analyzed by SYBR Green quantitative polymerase chain reaction (qPCR). For gene silencing, synovial sarcoma cells were transfected with small interfering RNAs (siRNAs) using Lipofectamine RNAiMAX according to the manufacturer’s protocol (Invitrogen). Cellular viability and migration activity were analyzed by the tetrazolium-based MTT and Boyden chamber assays, respectively. Three-dimensional tumor spheroids were generated from synovial sarcoma cells using a hanging-drop method (Foty, 2011) and cultured in ultra-low attachment microplates during drug treatment. Spheroid size was quantified with ImageJ. For cellular and molecular assays, statistically significant differences were determined by two-tailed Student’s *t*-test.

## Results

### Identification of SS18-SSX target genes in CRISPR-engineered synovial sarcoma cells

Lack of suitable antibodies detecting endogenous SS18-SSX fusion protein remains a major challenge in studying synovial sarcoma biology. We have recently used the CRISPR/Cas9 technology to insert a FLAG-tag sequence at the 3’ end of the *SSX2* gene in human SYO-1 cells (a well-established synovial sarcoma cell line) (Supplementary Figure S1A) (Patel et al., 2019). While the molecular mass of naïve *SSX2* gene product is about 22 kDa, the *SS18-SSX2* fusion produces a higher-molecular-weight protein of 75 kDa. In a western blot experiment, the anti-FLAG antibody only detected a 75 kDa band representing the SS18-SSX2 fusion protein in extracts from CRISPR-modified SSX2-FLAG SYO-1 cells (Supplementary Figure S1B, lanes 1-2). The identity of the 75 kDa protein band was further confirmed by anti-FLAG immunoprecipitation assay. Western blot analysis of the FLAG-SSX2 immunoprecipitates showed strong SS18 signal at 75 kDa (Supplementary Figure S1C), indicating that the peptide sequences of SS18 and SSX2 coexist in the FLAG-immunopurified protein. As an additional specificity control, we also tested parental (unmodified) SYO-1 cells and failed to detect any protein that efficiently cross-reacts with the anti-FLAG antibody on western blot (Supplementary Figure S1B, lanes 3-4). These results support the idea that SSX2-FLAG SYO-1 cells express endogenous SS18-SSX2 fusion protein carrying a C-terminal FLAG tag.

**FIGURE 1:**
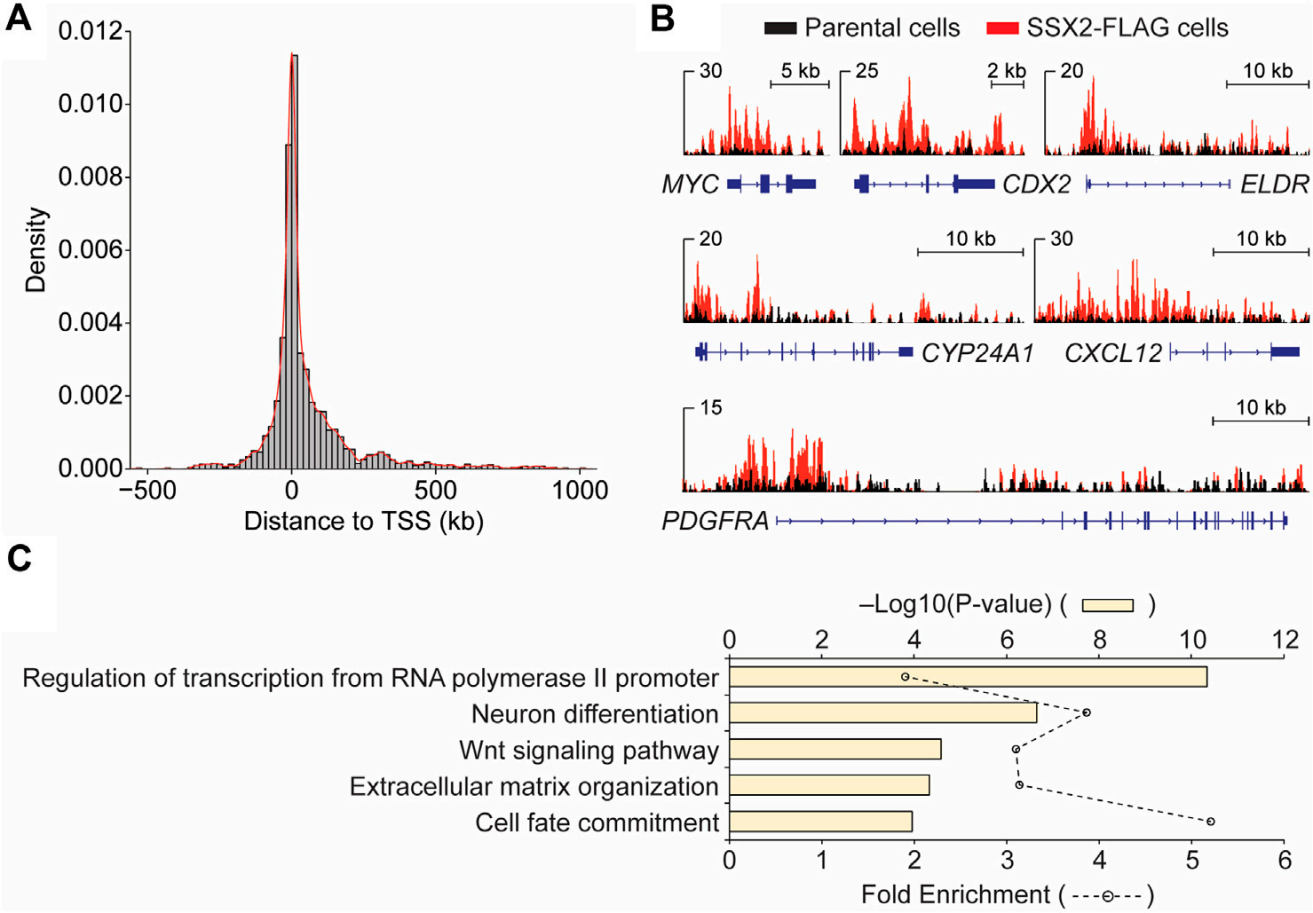
Genomic analysis of FLAG-SS18-SSX2 in CRISPR-modified synovial sarcoma cells. **(A)** Enrichment of FLAG-SS18-SSX2 binding sites around the transcription start site (TSS) in CRISPR-modified (SSX2-FLAG) SYO-1 cells. **(B)** Examples of IGV views of FLAG ChIP-seq in parental and SSX2-FLAG cells. **(C)** Gene ontology (GO) analysis of biological processes for FLAG ChIP-seq data.

Next, we examined the genomic distribution of SS18-SSX2 in SSX2-FLAG SYO-1 cells by chromatin immunoprecipitation sequencing (ChIP-seq). This analysis identified 3,172 FLAG peaks, with 12% at promoters, 49% at intragenic regions (including exons and introns) and 36% at intergenic regions (Supplementary Figure S2). A further examination of the peak distance to transcription start site (TSS) revealed that FLAG-tagged SS18-SSX2 primarily occupies genomic regions close to the TSS (Figure 1A). Previous studies have determined a direct role for the SS18-SSX fusion protein in regulating expression of several cancer-related genes, such as EGR1 (Lubieniecka et al., 2008), FOS (Su et al., 2012), IGF2 (de Bruijn et al., 2006; Sun et al., 2006), FZD10 (Tamaki et al., 2015), SOX2 (Kadoch and Crabtree, 2013), UNCX (Banito et al., 2018; Brien et al., 2018) and CDH4 (Boulay et al., 2021). Our ChIP-seq data not only confirmed the occupancy of SS18-SSX2 at known target gene regions (Supplementary Figure S3), but also uncovered new candidate SS18-SSX2 target genes, such as *MYC*, *CDX2* and *PDGFRA* (Figure 1B), known to be involved in the control of cell proliferation and transformation. Through a gene ontology (GO) analysis of FLAG ChIP-seq data, it became clear that SS18-SSX2 likely targets distinct sets of genes that are associated with specific biological processes (Figure 1C). This observation is reminiscent of the previously reported involvement of the SS18-SSX fusion protein in several fundamental cellular functions including transcriptional regulation (de Bruijn et al., 2007; Nacev et al., 2020), neurogenic differentiation (Ishibe et al., 2008; Banito et al., 2018), extracellular matrix remodeling (Saito, 2013), as well as WNT/beta-catenin signaling responsible for cell fate determination (Pretto et al., 2006; Trautmann et al., 2014; Cironi et al., 2016). Together, generation of SSX2-FLAG SYO-1 cells provides us with a unique opportunity to dissect endogenous SS18-SSX2 function and gain new insights into synovial sarcoma biology.

### SS18-SSX downregulates the proto-oncogene FYN in synovial sarcoma

**FIGURE 2:**
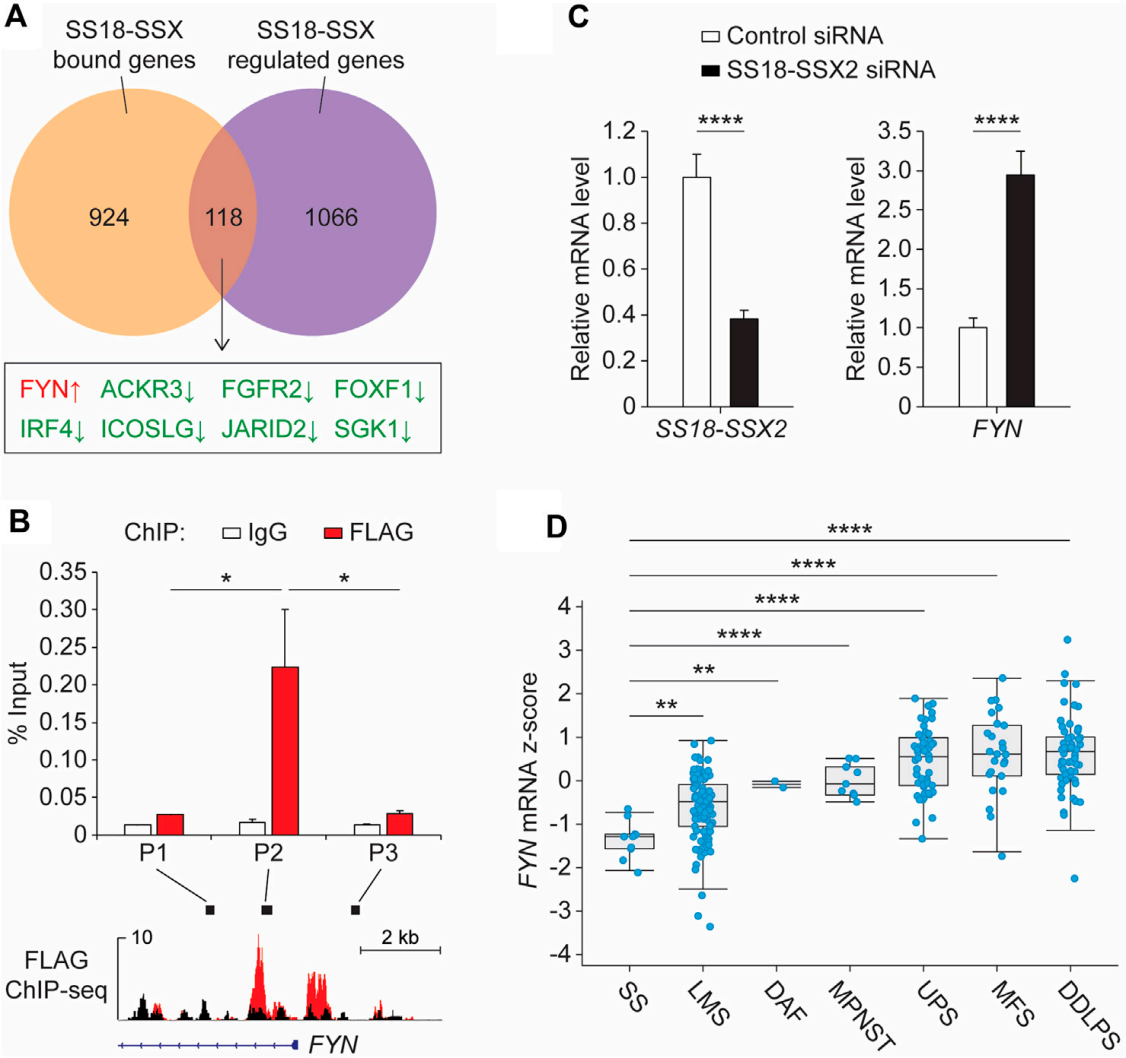
SS18-SSX2 represses FYN gene expression in synovial sarcoma cells. **(A)** Comparison of the SS18-SSX2 binding genes (detected by FLAG ChIP-seq) and the genes differentially expressed upon SS18-SSX2 knockdown (RNA-seq) in SYO-1 cells. **(B)** FLAG ChIP-qPCR analysis of SS18-SSX2 occupancy at the FYN gene locus in SYO-1 cells. **(C)** qPCR analysis of FYN mRNA levels in control and SS18-SSX2-knockdown SYO-1 cells. **(D)** FYN mRNA expression in soft tissue sarcoma samples from the TCGA database. SS, synovial sarcoma (n = 10); LMS, leiomyosarcoma (n = 99); DAF, desmoid/aggressive fibromatosis (n = 2); MPNST, malignant peripheral nerve sheath tumor (n = 9); UPS, undifferentiated pleomorphic sarcoma (n = 50); MFS, myxofibrosarcoma (n = 25); DDLPS, dedifferentiated liposarcoma (n = 58). ChIP-qPCR **(B)** and qPCR **(C)** data represent mean ± SD of three independent experiments. *p < 0.05; **p < 0.01; ****p < 0.0001 (two-tailed Student’s *t*-test).

The SS18-SSX fusion protein plays a critical role in both transcriptional activation and repression by interacting with different classes of epigenetic regulators (Nielsen et al., 2015; Hale et al., 2019). To test the integrity of the fusion protein complexes in SSX2-FLAG SYO-1 cells, SS18-SSX2 was first immunoprecipitated using the anti-FLAG antibody and then subjected to SDS-polyacrylamide gel electrophoresis. Western blot analysis of the immunopurified products confirmed the presence of BRG1, TLE1 and HDAC1 (Supplementary Figure S4), three major SS18-SSX-binding proteins seen previously. BRG1 is a core component of the mammalian switch/sucrose-nonfermentable (mSWI/SNF) complex required for SS18-SSX-mediated transcriptional activation (Kadoch and Crabtree, 2013; McBride et al., 2018), while TLE1 and HDAC1 form a Groucho corepressor complex to reduce transcription of several SS18-SSX target genes (Su et al., 2012; Cironi et al., 2016). In addition to immunoprecipitation analysis, a complementary genomic approach was employed to examine the functional impact of SS18-SSX2 on cancer gene expression (Supplementary Figure S5). We performed differential expression analysis of RNA sequencing (RNA-seq) data from SYO-1 cells transduced with a nonspecific (control) short-hairpin RNA (shRNA) or a *SSX2* shRNA targeting the *SS18-SSX2* fusion (Supplementary Figure S4) (McBride et al., 2018). A comparison of our ChIP-seq data with the above RNA-seq data revealed 118 overlapping genes (Figure 2A), which were recognized by SS18-SSX2 and whose expression was regulated by the fusion protein. When inspecting the OncoKB database (Chakravarty et al., 2017), we found eight overlapping genes falling into the class of oncogenes and, as expected, most of them (seven genes) being downregulated after *SS18-SSX2* knockdown (Figure 2A). *FYN* emerged as the only “oncogene” hit showing increased expression in the absence of SS18-SSX2 (Figure 2A and Supplementary Figure S6B). This observation prompted us to focus on *FYN* for further analysis, especially given that *FYN* encodes a protein kinase functionally linked to cancer cell survival, migration and drug resistance (Peng and Fu, 2023).

To provide a direct molecular link between the SS18-SSX2 fusion and *FYN* gene expression, we conducted anti-FLAG ChIP experiments in SSX2-FLAG SYO-1 cells. The precipitated DNA was analyzed by quantitative polymerase chain reaction (qPCR) with distinct primer sets adjacent to the *FYN* promoter region. A significant enrichment was observed at the area (P2) near the TSS of *FYN*, which is consistent with the pattern of SS18-SSX2 occupancy mapped by ChIP-seq assay (Figure 2B, red bars). As a specificity control, we examined the same *FYN* gene locus by ChIP-qPCR using the non-immune IgG antibody, but no signal was apparently enriched in the IgG-purified DNA product (Figure 2B, white bars). Having confirmed SS18-SSX2 association with the *FYN* gene, we next attempted to determine the role of SS18-SSX2 in regulating *FYN* transcription. Based on the RNA-seq data from shRNA-treated SYO-1 cells, it seems evident that *SS18-SSX2* knockdown by *SSX2* shRNA resulted in the induction of *FYN* gene expression (Supplementary Figure S6B). As an independent verification, we treated SYO-1 cells with a small interfering RNA (siRNA) duplex specifically targeting the fusion region of *SS18-SSX2* (Lubieniecka et al., 2008). After 2 days of incubation, *FYN* mRNA level was significantly increased in *SS18-SSX2*-depleted SYO-1 cells relative to the mock cells expressing a nonspecific control siRNA (Figure 2C). These results provide evidence for the involvement of SS18-SSX2 in downregulation of *FYN* gene expression. To examine the clinical relevance of this finding, we analyzed *FYN* expression in sarcoma patients (Hoadley et al., 2018) and found that *FYN* mRNA levels are significantly lower in synovial sarcoma than in any type of sarcomas that do not carry the SS18-SSX fusion (Figure 2D). It is worth noting that two *SSX* genes, namely, *SSX1* and *SSX2*, are found to commonly be fused to *SS18* in synovial sarcoma patients (Clark et al., 1994; Kawai et al., 1998; Ladanyi et al., 2002). In this regard, we compared the SS18-SSX2 cases with the SS18-SSX2 cases but observed no difference in *FYN* gene expression between the two fusion types (Supplementary Figure S7). Importantly, when compared to the genetic alterations of *SS18*, *SSX1* and *SSX2* alone (including amplification, deletion, point mutations and two gene fusions, *SS18-ZNF521* and *KDM6A-SSX1*), both SS18-SSX1 and SS18-SSX2 cases expressed significantly reduced levels of *FYN* mRNA (Supplementary Figure S7). Thus, it seems likely that *FYN* downregulation in synovial sarcoma is mediated by SS18-SSX regardless of its fusion type. In light of this, we performed siRNA-based knockdown assay in another human synovial sarcoma cell line (Yamato-SS) to target endogenous *SS18-SSX1* fusion gene. After depletion of *SS18-SSX1* from Yamato-SS cells, *FYN* mRNA level was significantly elevated (Supplementary Figure S8). This is consistent with the results obtained from SYO-1 cells, which showed that induction of *FYN* gene expression is correlated with the loss of SS18-SSX2 (Figure 2C). These observations together with the ChIP data suggest that SS18-SSX can bind to the *FYN* gene locus and repress its transcription in synovial sarcoma cells.

### FYN expression is derepressed in response to HDACi treatment

**FIGURE 3:**
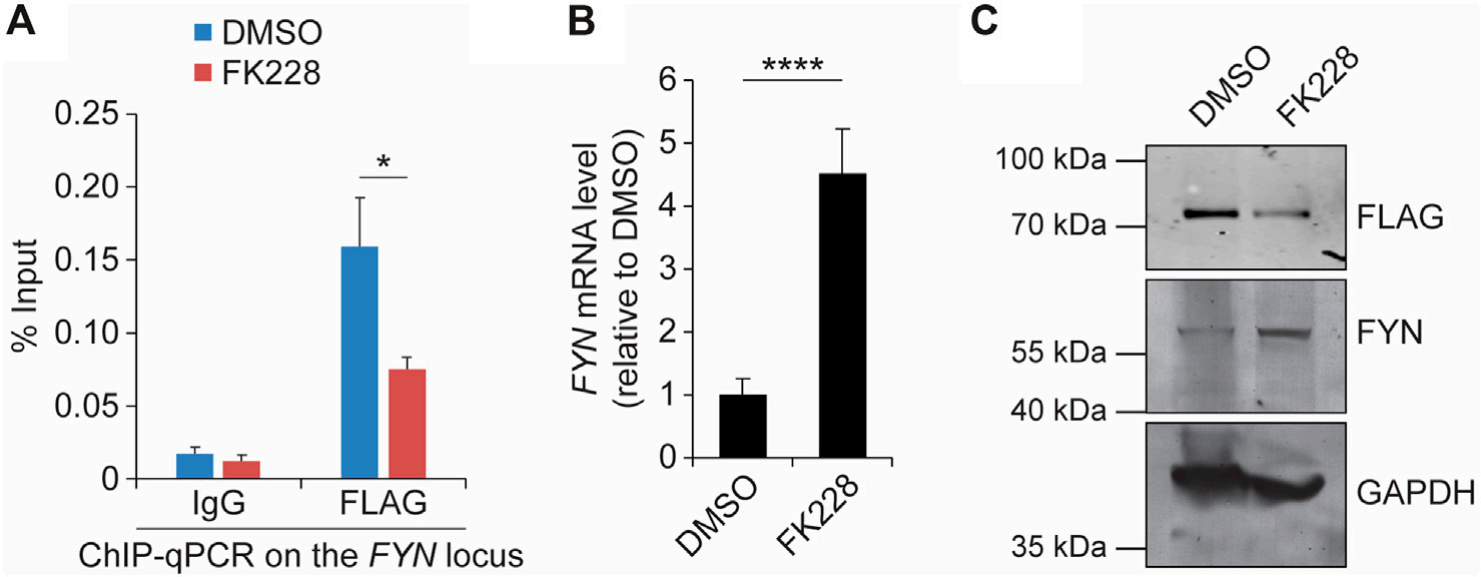
Suppression of SS18-SSX2 by FK228 induces FYN expression. **(A)** FLAG ChIP-qPCR analysis of SS18-SSX2 occupancy at the FYN gene locus before and after FK228 treatment. **(B)** qPCR analysis of FYN mRNA levels in DMSO- and FK228-treated SYO-1 cells. **(C)** Western blot analysis of SS18-SSX2 (anti-FLAG) and FYN protein levels in DMSO- and FK228-treated cells. GAPDH serves as the loading control. Data represent mean ± SD of three independent experiments. *p < 0.05; ****p < 0.0001 (two-tailed Student’s *t*-test).

To gain insight into the mechanism by which SS18-SSX represses *FYN* transcription, we tested the DNA-binding activity of TLE1 and HDC1, which are known to bind to the SS18-SSX fusion protein (Su et al., 2012; Cironi et al., 2016; Laporte et al., 2016) and to form a chromatin-modifying complex to inhibit histone acetylation for gene silencing (Chen et al., 1999; Choi et al., 1999; Brantjes et al., 2001). ChIP-qPCR revealed that both TLE1 and HDAC1 occupy the *FYN* promoter in SYO-1 cells (Supplementary Figure S9A), as does the SS18-SSX fusion protein. Examination of the histone modification status within the *FYN* promoter further showed that blocking HDAC activity by the small molecule FK228 (also known as depsipeptide or romidepsin) triggers a marked decrease in repressive histone H3 lysine 27 trimethylation (H3K27me3), whereas TLE1/HDAC1-independent H3 lysine four trimethylation (H3K4me3) levels remain unchanged (Supplementary Figure S9B). Consistent with changes in histone acetylation, which is mostly associated with transcriptional activation, exposure of SYO-1 cells to FK228 resulted in significant induction of *FYN* gene expression (Supplementary Figure S9C). In addition to maintenance of transcriptional repression, we previously found that HDAC activity is also essential for stabilization of SS18-SSX protein in synovial sarcoma cells (Patel et al., 2019). In agreement, we observed significantly decreased occupancy of SS18-SSX at the *FYN* promoter upon FK228 treatment for 18 h (Figure 3A). This is correlated with a dramatic increase in *FYN* mRNA level (Figure 3B). The expression levels of *FYN* in vehicle- and FK228-treated SYO-1 cells have also been validated by western blot assay in which 18-h exposure of FLAG-SSX2 cells to FK228 resulted in a major decrease in SS18-SSX2 level and increase in FYN protein abundance (Figure 3C). Similarly, addition of FK228 induced FYN transcription and protein expression in Yamato-SS cells (Supplementary Figure S10). These results underscore the importance of SS18-SSX function in mediating *FYN* repression and suggest inhibition of HDAC activity as a molecular mechanism of the switch to activation of *FYN* in synovial sarcoma cells.

### Inhibition of FYN induces synergy with HDACi treatment against synovial sarcoma

**FIGURE 4:**
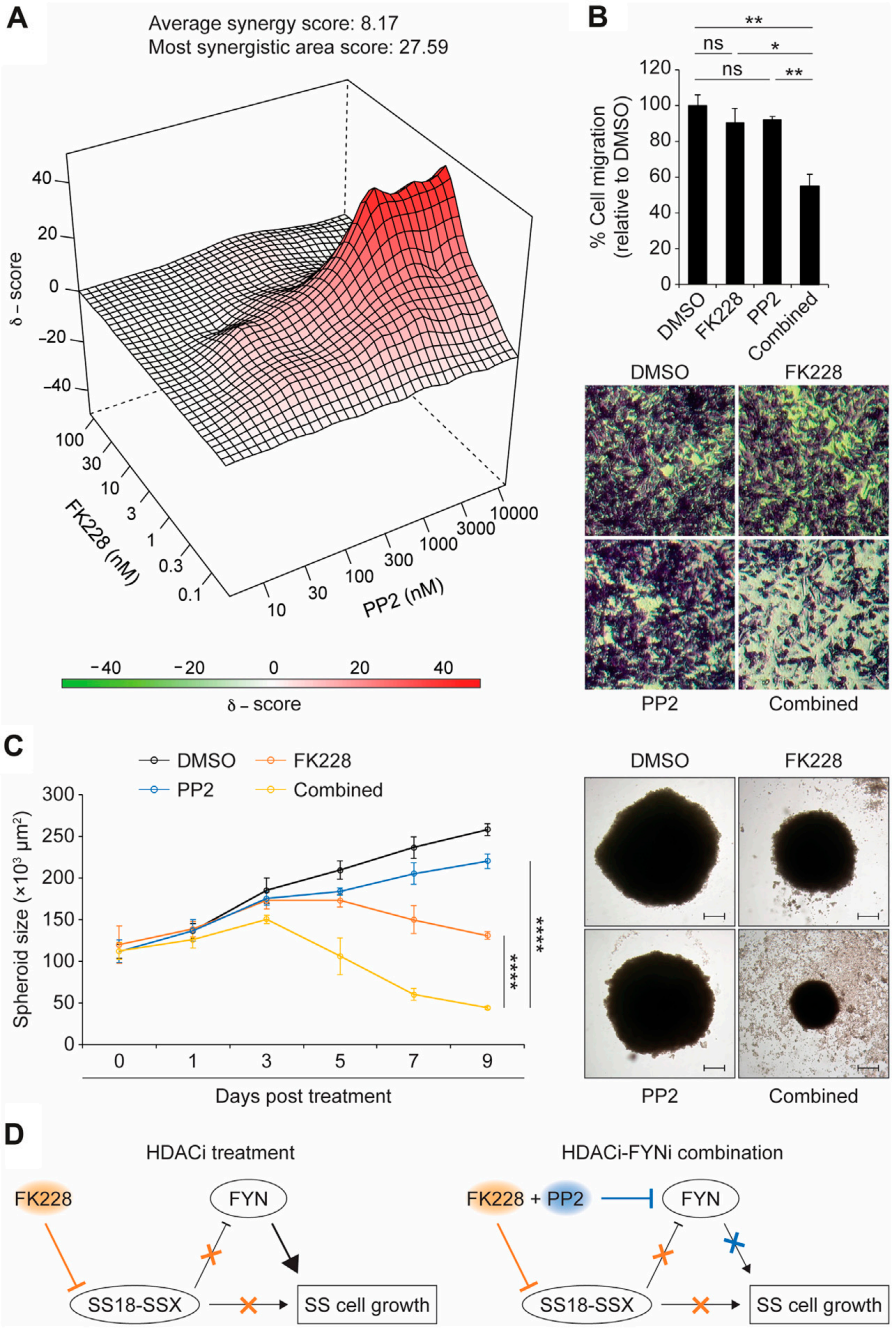
The FYN inhibitor PP2 synergistically enhances the efficacy of FK228 in synovial sarcoma cells. **(A)** 3D plot depicting a synergistic response between FK228 and PP2 in SYO-1 cells. **(B)** Changes in the migration of SYO-1 cells following exposure to either FK228 (0.01 μM) or PP2 (3 μM) alone or their combination. Migrated cells were stained with crystal violet (20× magnification). **(C)** Effects of FK228 and PP2 treatment alone or in combination on SYO-1 spheroid growth. Representative images of SYO-1 spheroids at day 7 in 3D culture (scale bars, 100 μm). **(D)** A model for synergy between HDAC and FYN inhibitors in synovial sarcoma treatment. Data represent mean ± SD of 3-4 independent experiments. *p < 0.05; **p < 0.01; ****p < 0.0001; ns, not significant (two-tailed Student’s *t*-test).

Although the biological function of FYN in synovial sarcoma remains unknown, previous studies have established a critical role for the FYN kinase in promoting cell proliferation and migration during cancer development. In addition, it has been reported that upregulation of *FYN* expression in mesothelioma and breast cancer cells stimulates significant resistance to anti-cancer agents by activation of anti-apoptotic and cell-cycle-regulatory proteins. These findings opened the question of whether FYN antagonizes the sensitivity of synovial sarcoma cells to HDACi treatment. To test this possibility, we first treated SYO-1 cells with the small molecule PP2, a SRC family kinase (SFK) inhibitor which preferentially blocks the action of FYN (Hanke et al., 1996). In a cell viability assay, addition of PP2 without the HDACi FK228 did not exhibit robust anti-synovial sarcoma effect (Supplementary Figure S11A), in keeping with the limited amount of FYN protein present in SYO-1 cells (Figure 3C). Next, PP2 was tested for its ability to modulate synovial sarcoma cell viability in the presence of FK228. While FK228 treatment alone clearly inhibited SYO-1 cell viability, the addition of PP2 to FK228 produced a markedly enhanced treatment effect (Supplementary Figure S11B), correlated with a significant reduction in half-maximal inhibitory concentration (IC50) values (Supplementary Figure S11C). To validate this observation more thoroughly, SYO-1 cells were treated with a series of FK228-PP2 combinations. Cell viability was measured 2 days after treatment to generate an 8 × 8 dose-response matrix. Using SynergyFinder (Ianevski et al., 2022), we detected a strong synergistic response (ZIP score >5) between FK228 and PP2 (Figure 4A). Importantly, this synergistic effect was not limited to SS18-SSX2-expressing SYO-1 cells, but was also observed in SS18-SSX1-expressing Yamato-SS cells as reflected by the results of viability, IC50 and SynergyFinder analyses (Supplementary Figure S12). These findings imply that FK228 induction of *FYN* expression likely plays a protective role in synovial sarcoma biology, and that blocking FYN activity by PP2 can improve the efficacy of FK228 in synovial sarcoma treatment.

Since FYN is known to promote metastasis in many types of cancer, we also asked whether FYN plays a similar role in synovial sarcoma. To this end, Boyden chamber assay was carried out to measure the transmembrane migratory activity of synovial sarcoma cells. We observed a clear decrease in the migration of both SYO-1 and Yamato-SS cells co-treated with FK228 and PP2, when compared to the DMSO control (Figure 4B and Supplementary Figure S13A). By contrast, treatment with FK228 or PP2 alone did not significantly alter SYO-1 and Yamato-SS cell migration behavior (Figure 4B and Supplementary Figure S13A). Therefore, at least in part, by blocking FYN activity, PP2 synergizes with FK228 to reduce synovial sarcoma cell migration.

To further explore the therapeutic potential of the FK228-PP2 combination, we used a hanging-drop method to generate three-dimensional (3D) tumor spheroids from SYO-1 and Yamato-SS cells. After spheroid establishment, FK228 and PP2 were added as either single agents or in combination. In keeping with the cell viability results, treatment with FK228 inhibited spheroid growth, whereas PP2 alone did not exert an obvious effect (Figure 4C and Supplementary Figure S13B). When PP2 was combined with FK228, we observed a more severe impairment in the growth of SYO-1 and Yamato-SS spheroids (Figure 4C and Supplementary Figure S13B). To extend our findings beyond the cell lines, we analyzed patient-derived synovial sarcoma cells (namely, SS18-SSX1-positive NCC-SS1 (Kito et al., 2018) and SS18-SSX2-positive NCC-SS2 (Oyama et al., 2018)) for FK228-PP2 combination treatment. Consistently, we found that PP2 alone does not have potent anti-synovial sarcoma activity (Supplementary Figure S14A, D). However, addition of PP2 to NCC-SS1 and NCC-SS2 cells could markedly improve the outcome of FK228 treatment, as reflected by increased drug sensitivity (Supplementary Figure S14B, E) and decreased IC50 doses (Supplementary Figure S14C, F). This effect was also observed in 3D culture, with more significant defects in NCC-SS1 and NCC-SS2 spheroid growth upon the combination of FK228 and PP2 than the single agents alone (Supplementary Figure S14G, H). Collectively, these observations reinforce the involvement of FYN in synovial sarcoma cell response to HDACi treatment, and suggest that inhibition of FYN activity may be a therapeutic strategy to enhance the effectiveness of HDACi treatment for synovial sarcoma.

## Discussion

A key step in the mechanism underlying synovial sarcoma is the fusion of SS18 and SSX genes. The resulting SS18-SSX fusion oncoprotein participates in both positive and negative regulation of gene expression, which subsequently leads to the activation of oncogenic pathways and the inhibition of tumour suppressor pathways (Nielsen et al., 2015). In this study, we identify the proto-oncogene FYN as a new direct target of SS18-SSX in human synovial sarcoma cells. While FYN is known to promote cellular proliferation and migration in multiple types of cancer (Peng and Fu, 2023), our findings reveal that SS18-SSX exerts a negative effect on FYN expression, at least in part, by repressing *FYN* transcription. This implies that FYN accumulation, following inhibition of SS18-SSX, could contribute to synovial sarcoma cell survival in a fusion oncogene-independent manner (Figure 4D, left).

Histone deacetylase 1 and 2 (HDAC1/2) have been implicated in regulation of SS18-SSX transcriptional activity and protein stability (Lubieniecka et al., 2008; Patel et al., 2019). We find that exposure of synovial sarcoma cells to FK228, a potent HDAC1/2 inhibitor (Furumai et al., 2002), leads to the induction of FYN mRNA and protein expression which could render synovial sarcoma cells resistant to growth inhibition after loss of SS18-SSX function. In keeping with this view, we show that combination of FK228 with the FYN inhibitor PP2 results in a synergistic treatment effect in synovial sarcoma cells (Figure 4D, right). Overall, our results potentially explain the clinical observation that HDACi treatment alone elicits limited anti-cancer activity in synovial sarcoma patients, and provide a biological rationale for blocking FYN function to improve HDACi action in synovial sarcoma treatment.

Finally, in terms of biological regulation and function of FYN in synovial sarcoma, some limitations should be considered. For example, despite a strong relationship between suppression of SS18-SSX and FYN derepression, we do not know whether there are other factors involved in this regulatory process. One of such factors could be EGR1, a transcription factor found in the *FYN* promoter to enhance FYN expression (Gao et al., 2009; Irwin et al., 2015). EGR1 is a direct target of SS18-SSX (de Bruijn et al., 2006; Lubieniecka et al., 2008) and its transcription level is negatively correlated with SS18-SSX activity in synovial sarcoma cells (Su et al., 2010; Laporte et al., 2016). It is possible that, in the absence of SS18-SSX or after HDACi treatment, increased amounts of EGR1 can facilitate activation of *FYN* transcription. Another limitation is that we present drug-response results from only established cell lines and primary cell culture, which may not fairly reflect the complex nature of original tumors. In this regard, further studies are still required to demonstrate the feasibility of FK228-PP2 combination strategy for treating synovial sarcoma in clinically relevant settings, such as human organoids and xenograft mouse models.

## Data Availability

The datasets presented in this study can be found in online repositories. The names of the repository/repositories and accession number(s) can be found in the article/Supplementary Material.

## References

[B1] Banito A. Li X. Laporte A. N. Roe J. S. Sanchez-Vega F. Huang C. H. (2018). The SS18-SSX oncoprotein hijacks KDM2B-PRC1.1 to drive synovial sarcoma. Cancer Cell 33 (3), 527–541. 10.1016/j.ccell.2018.01.018 29502955 PMC5881394

[B2] Benabdallah N. S. Dalal V. Scott R. W. Marcous F. Sotiriou A. Kommoss F. K. F. (2023). Aberrant gene activation in synovial sarcoma relies on SSX specificity and increased PRC1.1 stability. Nat. Struct. Mol. Biol. 30 (11), 1640–1652. 10.1038/s41594-023-01096-3 37735617 PMC10643139

[B3] Blay J. Y. von Mehren M. Jones R. L. Martin-Broto J. Stacchiotti S. Bauer S. (2023). Synovial sarcoma: characteristics, challenges, and evolving therapeutic strategies. ESMO Open 8 (5), 101618. 10.1016/j.esmoop.2023.101618 37625194 PMC10470271

[B4] Boulay G. Cironi L. Garcia S. P. Rengarajan S. Xing Y. H. Lee L. (2021). The chromatin landscape of primary synovial sarcoma organoids is linked to specific epigenetic mechanisms and dependencies. Life Sci. Alliance 4 (2), e202000808. 10.26508/lsa.202000808 33361335 PMC7768195

[B5] Brantjes H. Roose J. van De Wetering M. Clevers H. (2001). All Tcf HMG box transcription factors interact with Groucho-related co-repressors. Nucleic Acids Res. 29 (7), 1410–1419. 10.1093/nar/29.7.1410 11266540 PMC31284

[B6] Brett D. Whitehouse S. Antonson P. Shipley J. Cooper C. Goodwin G. (1997). The SYT protein involved in the t(X;18) synovial sarcoma translocation is a transcriptional activator localised in nuclear bodies. Hum. Mol. Genet. 6 (9), 1559–1564. 10.1093/hmg/6.9.1559 9285794

[B7] Brien G. L. Remillard D. Shi J. Hemming M. L. Chabon J. Wynne K. (2018). Targeted degradation of BRD9 reverses oncogenic gene expression in synovial sarcoma. Elife 7, e41305. 10.7554/eLife.41305 30431433 PMC6277197

[B8] Cassier P. A. Lefranc A. Amela E. Y. Chevreau C. Bui B. N. Lecesne A. (2013). A phase II trial of panobinostat in patients with advanced pretreated soft tissue sarcoma. A study from the French Sarcoma Group. Br. J. Cancer 109 (4), 909–914. 10.1038/bjc.2013.442 23922114 PMC3749588

[B9] Chakravarty D. Gao J. Phillips S. M. Kundra R. Zhang H. Wang J. (2017). OncoKB: a precision oncology knowledge base. JCO Precis. Oncol. 2017, 2017. 10.1200/PO.17.00011 PMC5586540 28890946

[B10] Chen G. Fernandez J. Mische S. Courey A. J. (1999). A functional interaction between the histone deacetylase Rpd3 and the corepressor groucho in Drosophila development. Genes Dev. 13 (17), 2218–2230. 10.1101/gad.13.17.2218 10485845 PMC316998

[B11] Choi C. Y. Kim Y. H. Kwon H. J. Kim Y. (1999). The homeodomain protein NK-3 recruits Groucho and a histone deacetylase complex to repress transcription. J. Biol. Chem. 274 (47), 33194–33197. 10.1074/jbc.274.47.33194 10559189

[B12] Chu Q. S. Nielsen T. O. Alcindor T. Gupta A. Endo M. Goytain A. (2015). A phase II study of SB939, a novel pan-histone deacetylase inhibitor, in patients with translocation-associated recurrent/metastatic sarcomas-NCIC-CTG IND 200†. Ann. Oncol. 26 (5), 973–981. 10.1093/annonc/mdv033 25632070

[B13] Cironi L. Petricevic T. Fernandes Vieira V. Provero P. Fusco C. Cornaz S. (2016). The fusion protein SS18-SSX1 employs core Wnt pathway transcription factors to induce a partial Wnt signature in synovial sarcoma. Sci. Rep. 6, 22113. 10.1038/srep22113 26905812 PMC4764983

[B14] Clark J. Rocques P. J. Crew A. J. Gill S. Shipley J. Chan A. M. (1994). Identification of novel genes, SYT and SSX, involved in the t(X;18)(p11.2;q11.2) translocation found in human synovial sarcoma. Nat. Genet. 7 (4), 502–508. 10.1038/ng0894-502 7951320

[B15] Crew A. J. Clark J. Fisher C. Gill S. Grimer R. Chand A. (1995). Fusion of SYT to two genes, SSX1 and SSX2, encoding proteins with homology to the Kruppel-associated box in human synovial sarcoma. EMBO J. 14 (10), 2333–2340. 10.1002/j.1460-2075.1995.tb07228.x 7539744 PMC398342

[B16] de Bruijn D. R. Allander S. V. van Dijk A. H. A. Willemse M. P. Thijssen J. van Groningen J. J. M. (2006). The synovial-sarcoma-associated SS18-SSX2 fusion protein induces epigenetic gene (de)regulation. Cancer Res. 66 (19), 9474–9482. 10.1158/0008-5472.CAN-05-3726 17018603

[B17] de Bruijn D. R. Nap J. P. van Kessel A. G. (2007). The (epi)genetics of human synovial sarcoma. Genes Chromosom. Cancer 46 (2), 107–117. 10.1002/gcc.20399 17117414

[B18] dos Santos N. R. de Bruijn D. R. van Kessel A. G. (2001). Molecular mechanisms underlying human synovial sarcoma development. Genes Chromosom. Cancer 30 (1), 1–14. 10.1002/1098-2264(2000)9999:9999<::aid-gcc1056>3.0.co;2-g 11107170

[B19] Foty R. (2011). A simple hanging drop cell culture protocol for generation of 3D spheroids. J. Vis. Exp. (51), 2720. 10.3791/2720 21587162 PMC3197119

[B20] Furumai R. Matsuyama A. Kobashi N. Lee K. H. Nishiyama M. Nakajima H. (2002). FK228 (depsipeptide) as a natural prodrug that inhibits class I histone deacetylases. Cancer Res. 62 (17), 4916–4921. 12208741

[B21] Gao Y. Howard A. Ban K. Chandra J. (2009). Oxidative stress promotes transcriptional up-regulation of Fyn in BCR-ABL1-expressing cells. J. Biol. Chem. 284 (11), 7114–7125. 10.1074/jbc.M804801200 19131339 PMC2652262

[B22] Haldar M. Hancock J. D. Coffin C. M. Lessnick S. L. Capecchi M. R. (2007). A conditional mouse model of synovial sarcoma: insights into a myogenic origin. Cancer Cell 11 (4), 375–388. 10.1016/j.ccr.2007.01.016 17418413

[B23] Haldar M. Hedberg M. L. Hockin M. F. Capecchi M. R. (2009). A CreER-based random induction strategy for modeling translocation-associated sarcomas in mice. Cancer Res. 69 (8), 3657–3664. 10.1158/0008-5472.CAN-08-4127 19351831 PMC2906130

[B24] Hale R. Sandakly S. Shipley J. Walters Z. (2019). Epigenetic targets in synovial sarcoma: a mini-review. Front. Oncol. 9, 1078. 10.3389/fonc.2019.01078 31681608 PMC6813544

[B25] Hanke J. H. Gardner J. P. Dow R. L. Changelian P. S. Brissette W. H. Weringer E. J. (1996). Discovery of a novel, potent, and Src family-selective tyrosine kinase inhibitor. Study of Lck- and FynT-dependent T cell activation. J. Biol. Chem. 271 (2), 695–701. 10.1074/jbc.271.2.695 8557675

[B26] Hoadley K. A. Yau C. Hinoue T. Wolf D. M. Lazar A. J. Drill E. (2018). Cell-of-Origin patterns dominate the molecular classification of 10,000 tumors from 33 types of cancer. Cell 173 (2), 291–304 e6. 10.1016/j.cell.2018.03.022 29625048 PMC5957518

[B27] Ianevski A. Giri A. K. Aittokallio T. (2022). SynergyFinder 3.0: an interactive analysis and consensus interpretation of multi-drug synergies across multiple samples. Nucleic Acids Res. 50 (W1), W739–W743. 10.1093/nar/gkac382 35580060 PMC9252834

[B28] Irwin M. E. Johnson B. P. Manshouri R. Amin H. M. Chandra J. (2015). A NOX2/Egr-1/Fyn pathway delineates new targets for TKI-resistant malignancies. Oncotarget 6 (27), 23631–23646. 10.18632/oncotarget.4604 26136341 PMC4695141

[B29] Ishibe T. Nakayama T. Aoyama T. Nakamura T. Toguchida J. (2008). Neuronal differentiation of synovial sarcoma and its therapeutic application. Clin. Orthop. Relat. Res. 466 (9), 2147–2155. 10.1007/s11999-008-0343-z 18563503 PMC2493002

[B30] Jones K. B. Barrott J. J. Xie M. Haldar M. Jin H. Zhu J. F. (2016). The impact of chromosomal translocation locus and fusion oncogene coding sequence in synovial sarcomagenesis. Oncogene 35 (38), 5021–5032. 10.1038/onc.2016.38 26947017 PMC5014712

[B31] Kadoch C. Crabtree G. R. (2013). Reversible disruption of mSWI/SNF (BAF) complexes by the SS18-SSX oncogenic fusion in synovial sarcoma. Cell 153 (1), 71–85. 10.1016/j.cell.2013.02.036 23540691 PMC3655887

[B32] Kawai A. Woodruff J. Healey J. H. Brennan M. F. Antonescu C. R. Ladanyi M. (1998). SYT-SSX gene fusion as a determinant of morphology and prognosis in synovial sarcoma. N. Engl. J. Med. 338 (3), 153–160. 10.1056/NEJM199801153380303 9428816

[B33] Kito F. Oyama R. Takai Y. Sakumoto M. Shiozawa K. Qiao Z. (2018). Establishment and characterization of the NCC-SS1-C1 synovial sarcoma cell line. Hum. Cell 31 (2), 167–174. 10.1007/s13577-018-0199-9 29450702

[B34] Krieg A. H. Hefti F. Speth B. M. Jundt G. Guillou L. Exner U. G. (2011). Synovial sarcomas usually metastasize after >5 years: a multicenter retrospective analysis with minimum follow-up of 10 years for survivors. Ann. Oncol. 22 (2), 458–467. 10.1093/annonc/mdq394 20716627

[B35] Ladanyi M. (2001). Fusions of the SYT and SSX genes in synovial sarcoma. Oncogene 20 (40), 5755–5762. 10.1038/sj.onc.1204601 11607825

[B36] Ladanyi M. Antonescu C. R. Leung D. H. Woodruff J. M. Kawai A. Healey J. H. (2002). Impact of SYT-SSX fusion type on the clinical behavior of synovial sarcoma: a multi-institutional retrospective study of 243 patients. Cancer Res. 62 (1), 135–140. 11782370

[B37] Laporte A. N. Ji J. X. Ma L. Nielsen T. O. Brodin B. A. (2016). Identification of cytotoxic agents disrupting synovial sarcoma oncoprotein interactions by proximity ligation assay. Oncotarget 7 (23), 34384–34394. 10.18632/oncotarget.8882 27120803 PMC5085163

[B38] Li C. Krasniqi F. Donners R. Kettelhack C. Krieg A. H. (2024). Synovial sarcoma: the misdiagnosed sarcoma. EFORT Open Rev. 9 (3), 190–201. 10.1530/EOR-23-0193 38457918 PMC10958242

[B39] Lubieniecka J. M. de Bruijn D. R. H. Su L. van Dijk A. H. A. Subramanian S. van de Rijn M. (2008). Histone deacetylase inhibitors reverse SS18-SSX-mediated polycomb silencing of the tumor suppressor early growth response 1 in synovial sarcoma. Cancer Res. 68 (11), 4303–4310. 10.1158/0008-5472.CAN-08-0092 18519690

[B40] McBride M. J. Pulice J. L. Beird H. C. Ingram D. R. D’Avino A. R. Shern J. F. (2018). The SS18-SSX fusion oncoprotein hijacks BAF complex targeting and function to drive synovial sarcoma. Cancer Cell 33 (6), 1128–1141. 10.1016/j.ccell.2018.05.002 29861296 PMC6791822

[B41] Nacev B. A. Jones K. B. Intlekofer A. M. Yu J. S. E. Allis C. D. Tap W. D. (2020). The epigenomics of sarcoma. Nat. Rev. Cancer 20 (10), 608–623. 10.1038/s41568-020-0288-4 32782366 PMC8380451

[B42] Nielsen T. O. Poulin N. M. Ladanyi M. (2015). Synovial sarcoma: recent discoveries as a roadmap to new avenues for therapy. Cancer Discov. 5 (2), 124–134. 10.1158/2159-8290.CD-14-1246 25614489 PMC4320664

[B43] Oyama R. Kito F. Sakumoto M. Shiozawa K. Toki S. Endo M. (2018). Establishment and proteomic characterization of a novel synovial sarcoma cell line, NCC-SS2-C1. Vitro Cell Dev. Biol. Anim. 54 (5), 392–399. 10.1007/s11626-018-0237-7 29626278

[B44] Patel N. Wang J. Shiozawa K. Jones K. B. Zhang Y. Prokop J. W. (2019). HDAC2 regulates site-specific acetylation of MDM2 and its ubiquitination signaling in tumor suppression. iScience 13, 43–54. 10.1016/j.isci.2019.02.008 30818224 PMC6393697

[B45] Peng S. Fu Y. (2023). FYN: emerging biological roles and potential therapeutic targets in cancer. J. Transl. Med. 21 (1), 84. 10.1186/s12967-023-03930-0 36740671 PMC9901160

[B46] Pretto D. Barco R. Rivera J. Neel N. Gustavson M. D. Eid J. E. (2006). The synovial sarcoma translocation protein SYT-SSX2 recruits beta-catenin to the nucleus and associates with it in an active complex. Oncogene 25 (26), 3661–3669. 10.1038/sj.onc.1209413 16462762

[B47] Rajwanshi A. Srinivas R. Upasana G. (2009). Malignant small round cell tumors. J. Cytol. 26 (1), 1–10. 10.4103/0970-9371.54861 21938141 PMC3167982

[B48] Saito T. (2013). The SYT-SSX fusion protein and histological epithelial differentiation in synovial sarcoma: relationship with extracellular matrix remodeling. Int. J. Clin. Exp. Pathol. 6 (11), 2272–2279. 24228088 PMC3816795

[B49] Su L. Cheng H. Sampaio A. V. Nielsen T. O. Underhill T. M. (2010). EGR1 reactivation by histone deacetylase inhibitors promotes synovial sarcoma cell death through the PTEN tumor suppressor. Oncogene 29 (30), 4352–4361. 10.1038/onc.2010.204 20514024

[B50] Su L. Sampaio A. V. Jones K. B. Pacheco M. Goytain A. Lin S. (2012). Deconstruction of the SS18-SSX fusion oncoprotein complex: insights into disease etiology and therapeutics. Cancer Cell 21 (3), 333–347. 10.1016/j.ccr.2012.01.010 22439931 PMC3734954

[B51] Sultan I. Rodriguez-Galindo C. Saab R. Yasir S. Casanova M. Ferrari A. (2009). Comparing children and adults with synovial sarcoma in the Surveillance, Epidemiology, and End Results program, 1983 to 2005: an analysis of 1268 patients. Cancer 115 (15), 3537–3547. 10.1002/cncr.24424 19514087

[B52] Sun Y. Gao D. Liu Y. Huang J. Lessnick S. Tanaka S. (2006). IGF2 is critical for tumorigenesis by synovial sarcoma oncoprotein SYT-SSX1. Oncogene 25 (7), 1042–1052. 10.1038/sj.onc.1209143 16247461

[B53] Tamaki S. Fukuta M. Sekiguchi K. Jin Y. Nagata S. Hayakawa K. (2015). SS18-SSX, the oncogenic fusion protein in synovial sarcoma, is a cellular context-dependent epigenetic modifier. PLoS One 10 (11), e0142991. 10.1371/journal.pone.0142991 26571495 PMC4646489

[B54] Thaete C. Brett D. Monaghan P. Whitehouse S. Rennie G. Rayner E. (1999). Functional domains of the SYT and SYT-SSX synovial sarcoma translocation proteins and co-localization with the SNF protein BRM in the nucleus. Hum. Mol. Genet. 8 (4), 585–591. 10.1093/hmg/8.4.585 10072425

[B55] Trautmann M. Sievers E. Aretz S. Kindler D. Michels S. Friedrichs N. (2014). SS18-SSX fusion protein-induced Wnt/β-catenin signaling is a therapeutic target in synovial sarcoma. Oncogene 33 (42), 5006–5016. 10.1038/onc.2013.443 24166495

[B56] Vlenterie M. Litière S. Rizzo E. Marréaud S. Judson I. Gelderblom H. (2016). Outcome of chemotherapy in advanced synovial sarcoma patients: review of 15 clinical trials from the European Organisation for Research and Treatment of Cancer Soft Tissue and Bone Sarcoma Group; setting a new landmark for studies in this entity. Eur. J. Cancer 58, 62–72. 10.1016/j.ejca.2016.02.002 26968015

