# Supplementary material for: Combination of HDAC and FYN inhibitors in synovial sarcoma treatment
Source: Front Cell Dev Biol. 2024 Jul 9;12:1422452. doi: 10.3389/fcell.2024.1422452 (PMC11264242; doi:10.3389/fcell.2024.1422452)
Supplement: Supplementary file 1 [file Presentation1.pdf]

Supplementary Material

Combination of HDAC and FYN inhibitors in synovial sarcoma treatment

Kyra Parker, Yanfeng Zhang, Gavin M. Anchondo, Ashlyn R. Smith, Sergio E. Guerrero Pacheco, Tadashi Kondo, Le Su

Materials and Methods

Cell culture and chemicals

Human synovial sarcoma cell lines SYO-1 (National Cancer Center, Japan) and Yamato-SS (Osaka Medical Center for Cancer and Cardiovascular Diseases, Japan) were cultured in DMEM (Invitrogen), supplemented with 10% fetal bovine serum (FBS), and grown at 37°C, 95% humidity and 5% CO2. All chemicals used in this study are commercially available: crystal violet (VWR Life Science), dimethyl sulfoxide (DMSO, Sigma), FK228 (S3020, Selleck Chemicals) and PP2 (S7008, Selleck Chemicals).

Establishment of patient-derived synovial sarcoma cells

Surgical excision specimens were collected from 72- and 42-year-old synovial sarcoma patients bearing the *SS18-SSX1* and *SS18-SSX2* fusion oncogenes, respectively. The tumor tissues were minced into small pieces and incubated with Collagenase II (Worthington) at 37°C for 15 min. After passing through 18-gauge needles and 40-μm cell strainers (Falcon), the cell suspension was cultured in DMEM supplemented with 10% FBS. Sanger sequencing was used to determine gene fusion events in primary cell cultures, namely *SS18-SSX1*-positive NCC-SS1 and *SS18-SSX2*-positive NCC-SS2 cells. All human studies were approved by the ethics committee of the National Cancer Center, Japan.

Immunoprecipitation and western blot

For immunoprecipitation, cell lysates were prepared using Pierce IP Lysis Buffer (Thermo Scientific) with protease inhibitor cocktails (Sigma). Equal amounts of cell lysates were incubated with 75 μL of Protein-A/G magnetic beads (Bio-Rad) coupled with 3 μg of the FLAG/M2 antibody (Sigma). After overnight rotation at 4°C, the beads were subjected to two washes with the IP buffer and two washes with PBS/T (0.1% Tween-20). Immunoprecipitates were eluted in 2× Bio-Rad Laemmli buffer at 95°C for 5 min. Whole cell lysis was prepared using RIPA Lysis Buffer (Santa Cruz Biotechnology), and protein concentrations were measured by Pierce Bradford Plus Protein assay (Thermo). For western blot assay, protein samples were separated on 10% sodium dodecyl sulfate-polyacrylamide gel electrophoresis (SDS-PAGE) and transferred to 0.45 μm nitrocellulose membranes (Bio-Rad). Blots were incubated with the primary antibodies against FLAG (M2, Sigma), SS18 (21792, Cell Signaling), BRG1 (49360, Cell Signaling), HDAC1 (PA1-860, Thermo), TLE1 (A303-545A, Bethyl Labs), FYN (4023, Cell Signaling), Tubulin (32293, Santa Cruz Biotechnology) and GAPDH (PA1-16777, Thermo), and visualized using BCIP/NBT substrate (Sigma).

Real-time qPCR

Total RNA was isolated and then transcribed to cDNA using the Qiagen RNeasy kit and the high-capacity cDNA reverse transcription kit (Applied Biosystems), respectively. SYBR Green qPCR assay (Applied Biosystems) was performed on QuantStudio Real-Time PCR System with specific primer sets: FYN (5-GGGTGCTAATGTGGAGACTG-3'; 5'-GCTTTGATGCTGACTTGCAG-3'), SS18-SSX1 (5'-CCAGCAGAGGCCTTATGGAT-3'; 5'-GGTGCAGTTGTTTCCCATCG-3'), SS18-SSX2 (5'-TGACCAGATCATGCCCAAG-3'; 5'-GGGTCCAGATCTCTCGTGAA-3'), and ACTIN (5'-TGCGTCTGGACCTGGCTGGC-3'; 5'-GCCTCAGGGCAGCGGAACCG-3'). All transcripts were normalized to ACTIN mRNA expression levels.

Chromatin Immunoprecipitation (ChIP)

ChIP experiments were performed using ChIP-IT Express Kit (Active Motif). Briefly, 2×10^7 cells were cross-linked with 1% formaldehyde prior to lysis. Cross-linked DNA was sheared into 200 to 400 bp using a Bioruptor sonicator (Diagenode). After centrifugation, the supernatants were pre-cleared with Protein G beads for 30 min at 4°C, and incubated with the indicated antibody at 4°C overnight. After 4-hr incubation with Protein G beads, the precipitates were washed four times with different washing buffers (Active Motif), eluted with 1% SDS, and incubated at 65°C overnight to reverse cross-linking. ChIP DNA was purified using Active Motif Purification Kit and subjected to SYBR Green qPCR using following primer sets: P1 (5'-ACAACATCACTGGGCAGACAGGAT-3'; 5'- TTGATAAGAGGGAGGCAGCCTGAA -3'), P2 (5'- AGGGAAGCAGCACGGG -3'; 5'-ATTTCCTCGCCCGCAAGC -3') and P3 (5'- TACAGTTGGGAGTGCATCGGTCTT -3'; 5'-AAATCAGGAAGCCAAAGTGTTTCT -3').

RNA interference (RNAi)

Small interfering RNAs (siRNAs) were purchased from Integrated DNA Technologies, for SS18-SSX2 (CAAGAAGCCAGCAGAGGAATT, UUCCUCUGCUGGCUUCUUGTT) and SS18-SSX1 (GAAAGCAGCUGGUGAUUUAUGAAGAAG, UCUUCAUAAAUCACCAGCUGCUUUCAU). Control siRNA was purchased from Santa Cruz Biotechnology (#37003). At 60% confluence in 6-well plates, cells were transfected with indicated siRNAs using Lipofectamine RNAiMAX according to the manufacturer's protocol (Invitrogen). Briefly, siRNAs were diluted in 200 µL of Opti-MEM Reduced Serum Medium (Invitrogen) at a final concentration of 60 nM and mixed gently with 7 µL of Lipofectamine RNAiMAX. After 5-minute incubation at room temperature, the siRNA-RNAiMAX reagent was added to cells for 48 hours at 37°C. The efficiency of SS18-SSX knockdown and the expression of FYN mRNA were confirmed by real-time qPCR analysis.

MTT assay

Human synovial sarcoma cells were seeded into 96-well plates at the density of 10^4 cells per well, and cultured overnight at 37°C. To test drug effects, cell viability was examined at the 48-hr time point after treatment. Cell cultures were incubated with the 3-(4,5-dimethylthiazol-2-yl)-2,5-diphenyltetrazolium bromide (MTT) solution (60 min at 37°C), and MTT formazan was solubilized in DMSO (10 min at room temperature). Absorbance was read at 570 nm with a colorimeter. Relative viability of drug-treated cells was calculated in comparison with control cells.

Boyden chamber assay

Cell migration was tested using 24-well cell culture Boyden chambers (CELLTREAT). Cells (3×10^5 per well) were plated in upper inserts with membranes containing 8 µm pores. After 24 hours of

treatment, cells that passed through the membrane were stained by 0.5% crystal violet and solubilized in DMSO for colorimetric detection (at 560 nm). Values were normalized to the vehicle control.

3D spheroid assay

Tumor spheroids were generated from SYO-1 and Yamato-SS cells using the hanging-drop method (2000 cells per 20 μL per drop). After 5 days of incubation, the spheroid droplets were transferred into 96-well ultra-low attachment microplates (Corning) containing 150 μL of media per well. Spheroids were treated with DMSO or FK228 for 9 days and photographed in an inverted microscope on the indicated days. Spheroid size was then measured by ImageJ at a micrometer scale.

Bioinformatic analysis

The raw ChIP-seq data (FASTQ format) were first adaptor-trimmed, then mapped to the human reference genome (hg19) using Bowtie2 program (version 2.1.0) with the default setting. After removing duplicated reads, we used the MACS2 (version 2.1) software to identify peaks using the matched DNA input data as the control. Based on the coordinate of called peaks, the overlap rate between two TF binding peaks was analyzed using the Bedtools program. The peaks were ranked by the number of mapped reads within the peak interval and the top 10% of peaks were selected for motif discovery. The summits of the top 10% peaks were extended by 100 bp on either side. Motifs between 5 and 30 bp in length were identified on both strands. We employed the MEME 4.9.1 toolkit to search DNA motifs and enrichment significance for candidate TFs.

We processed RNA-seq data (GSE108028) as follows. After trimming the 5' and 3' adaptors and eliminating contaminants and inadequate (<20 nt) and low-quality reads using cutadapt (v 2.10), all clean sequencing data were mapped to the human reference genome (hg19) using Hisat2 program (v 2.1.0) with the default setting. Aligned data in SAM format were processed and converted into BAM files using SAMtools program. To quantify gene expression levels, read counts were calculated using the featureCounts (version 2.0.2) program, then implemented in the edgeR package to calculate the count per million (CPM) values. The differential expression (DE) analyses were performed using edgeR implemented in the Bioconductor package to identify differentially expressed genes between experiments under different conditions.

Statistical analysis

All experiments were carried out three-to-four independent times. Results were analyzed using the GraphPad Prism software and presented as mean ± standard deviation (SD). For comparison between two experimental groups, statistics was performed using two-tailed Student's *t*-test, and P value < 0.05 was considered significant.

Supplementary Figures

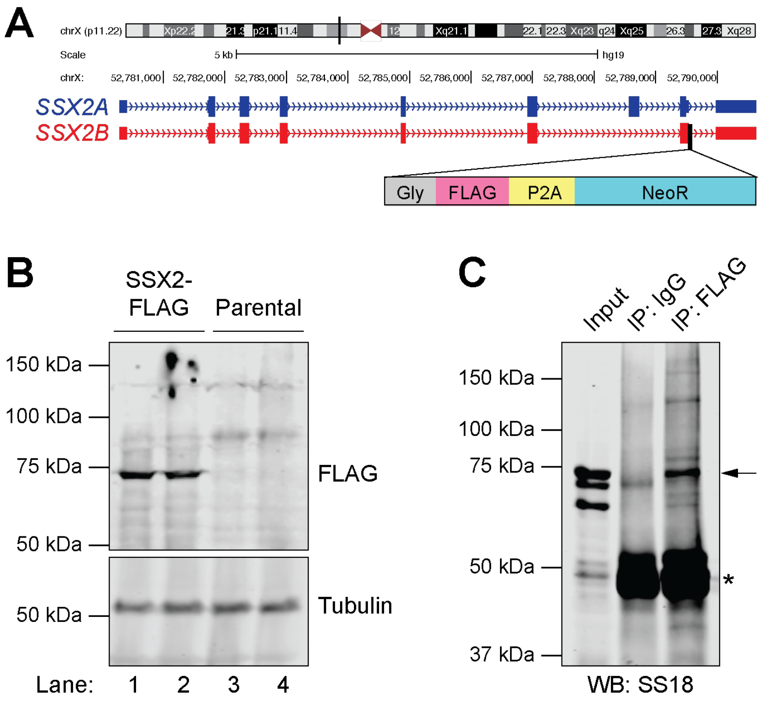

Figure S1. Characterization of SSX2-FLAG SYO-1 cells.

(**A**) Insertion of a FLAG tag into the SSX2B gene locus by CRISPR. (**B**) Western blot analysis of whole cell lysates prepared from parental and CRISPR-modified SYO-1 cells. Tubulin serves as the loading control. (**C**) Western blot analysis of FLAG immunoprecipitates with the SS18 antibody. Arrow indicates the position of the SS18-SSX fusion protein, and asterisk indicates IgG.

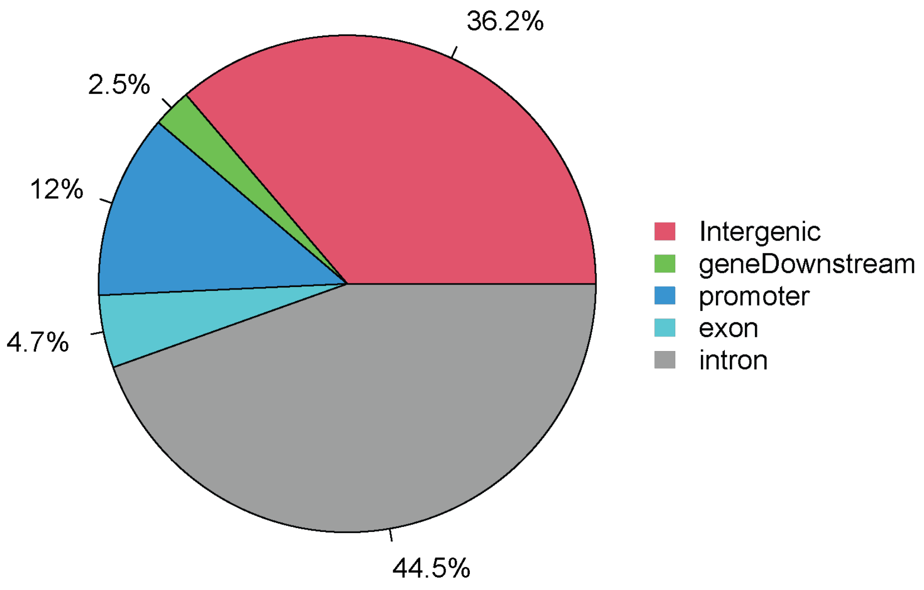

Figure S2. Pie chart depicting the genomic distribution of SS18-SSX2 binding sites detected by FLAG ChIP-seq analysis.

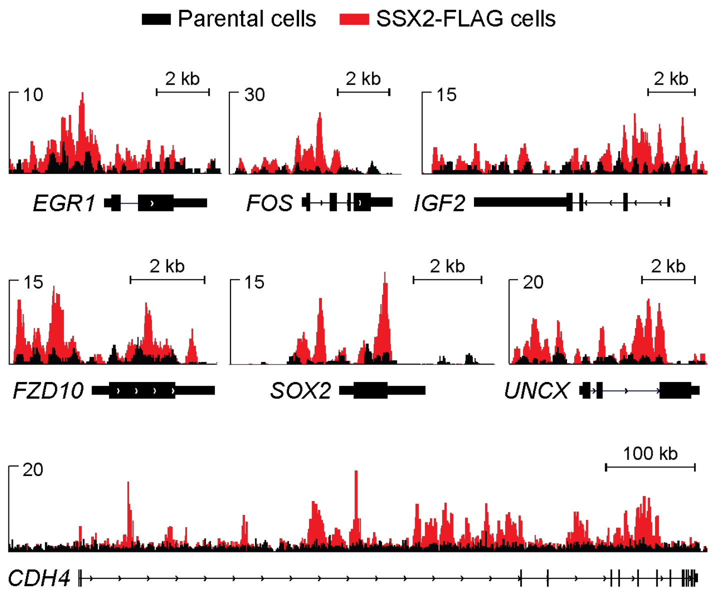

Figure S3. IGV views showing the occupancy of FLAG-SS18-SSX2 at its known target gene loci. Parental SYO-1 cells were used to account for the background signal.

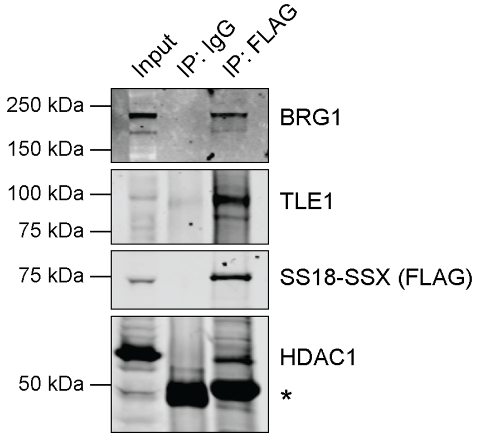

Figure S4. FLAG immunoprecipitation assay showing the interaction of SS18-SSX with its known cofactors in SYO-1 cells. Input and IgG serve as the positive and negative controls, respectively. Asterisk indicates the IgG bands.

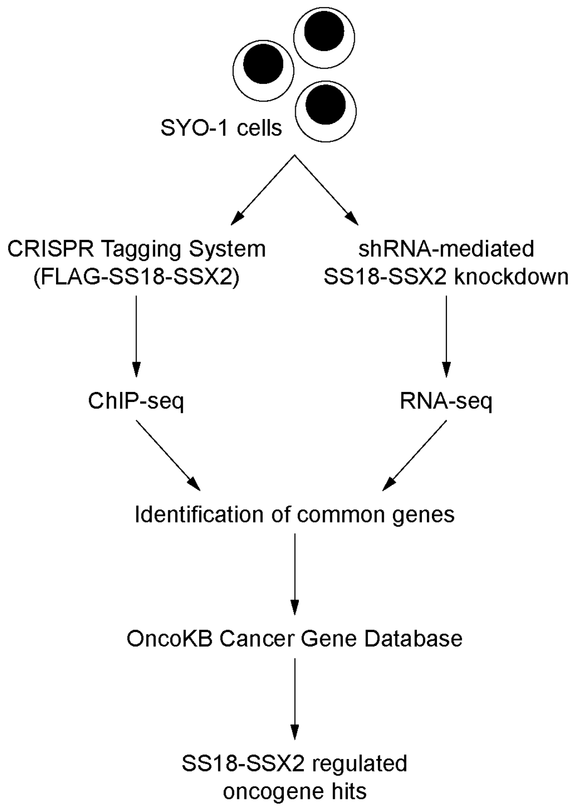

Figure S5. Schematic of the integration of ChIP-seq and RNA-seq datasets from SYO-1 cells to elucidate SS18-SSX-dependent oncogene regulation.

A

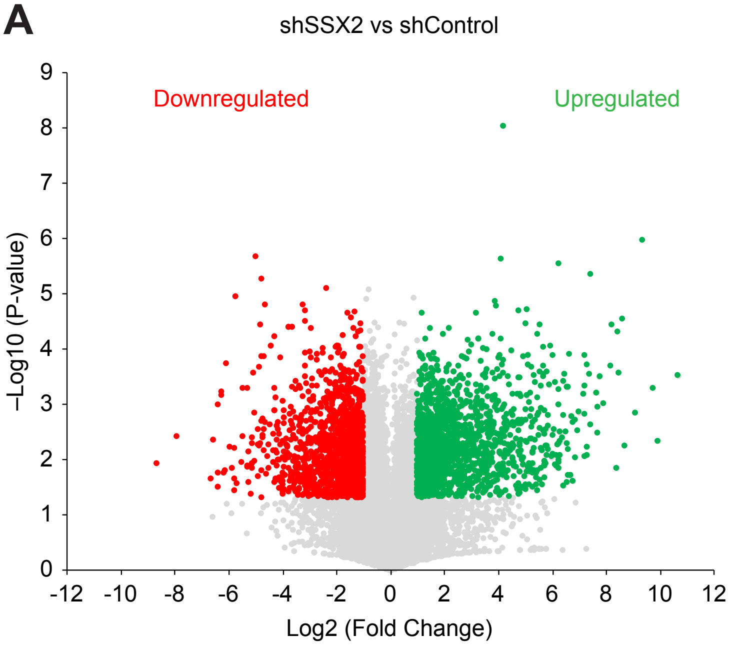

B

Gene Name	Log_2FC	P-value
FYN	2.07430734	0.00000322
ACKR3	-2.715876195	8.75018E-64
FGFR2	-3.986544372	1.01842E-17
FOXF1	-1.342953896	2.29627E-06
IRF4	-3.552325035	1.68533E-08
ICOSLG	-2.821025729	0.00271448
JARID2	-1.811878264	0.00037957
SGK1	-1.106641542	3.26312E-07

Figure S6. RNA-seq analysis of untreated and shSSX2-treated SYO-1 cells.

(**A**) Examination of transcription profiles between control and shSSX2-expressing SYO-1 cells uncovered 1,184 differentially expressed genes (fold change > 2 and adjusted P-value < 0.05). Upon shRNA-mediated SS18-SSX2 depletion, there were 801 up- and 383 down-regulated genes colored in green and red, respectively. (**B**) A list of SS18-SSX2-binding proto-oncogenes whose expression was significantly changed after SS18-SSX2 knockdown in SYO-1 cells.

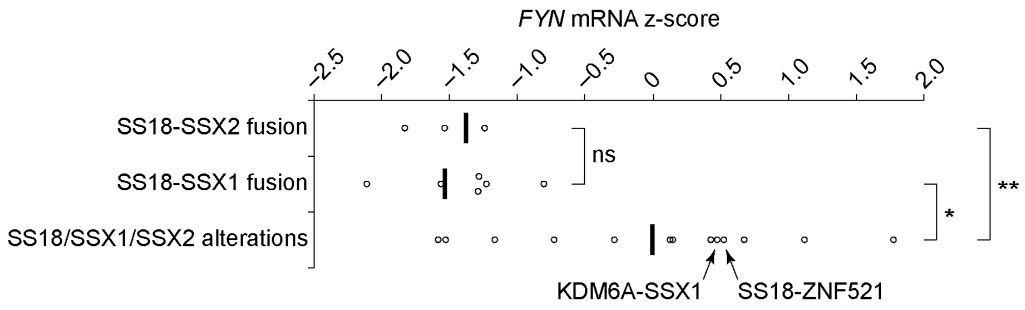

Figure S7. Comparison of FYN mRNA expression levels in sarcoma patients associated with SS18-SSX1, SS18-SSX2 and other SS18/SSX1/SSX2-related genetic alterations. *$P < 0.05$; **$P < 0.01$; ns, not significant (two-tailed Student's *t*-test).

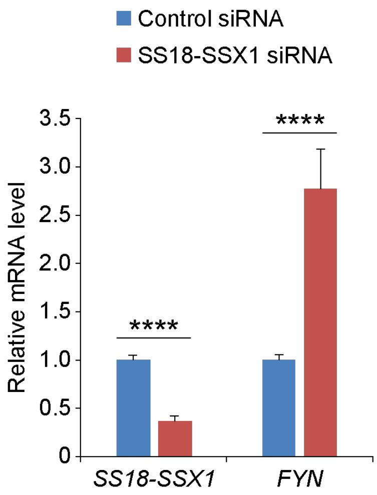

Figure S8. Effect of SS18-SSX1 knockdown on FYN mRNA expression in Yamato-SS cells. Data represent mean ± SD of three independent experiments; ****$P < 0.0001$, determined by two-tailed Student's *t*-test.

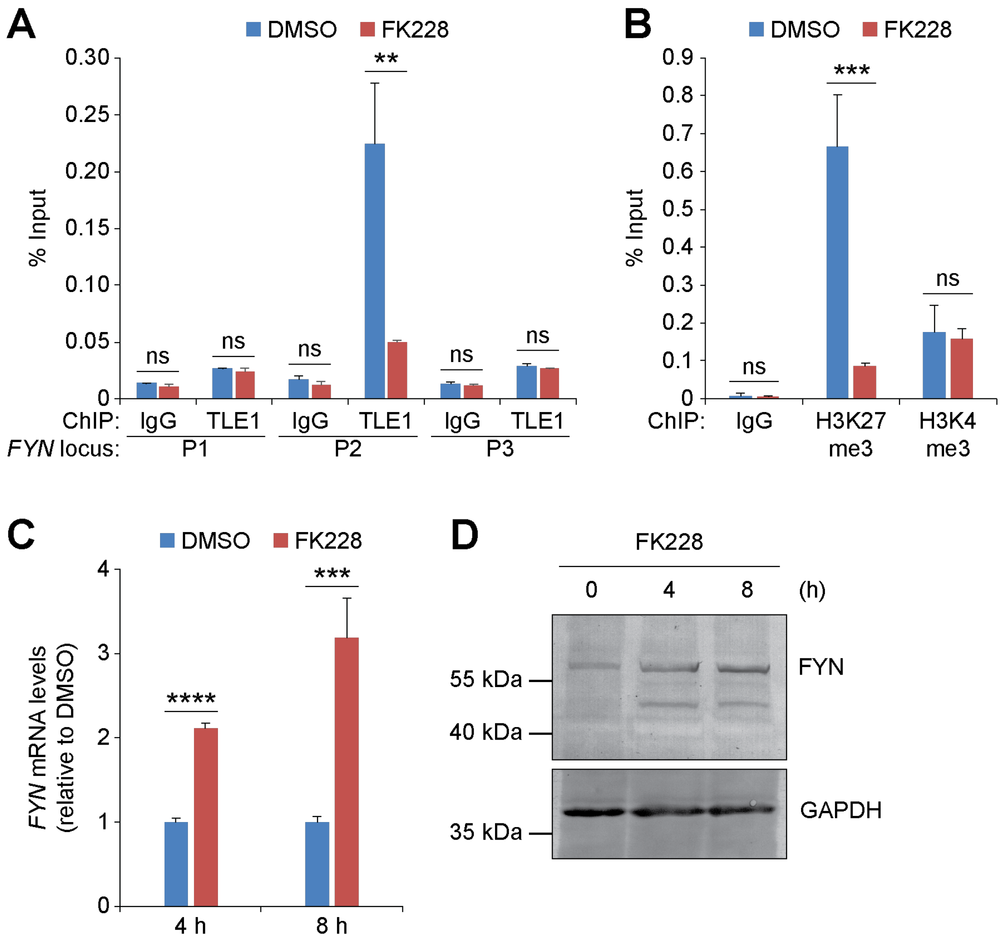

Figure S9. Effect of FK228 treatment on FYN expression in SYO-1 cells.

(**A**) Reduction of TLE1 recruitment to the FYN gene locus in SYO-1 cells treated with FK228. (**B**) Reduction of repressive H3K27me3 mark at the FYN gene locus in SYO-1 cells treated with FK228. (**C**) Induction of FYN mRNA expression in SYO-1 cells following the 4-8 h treatment of FK228 (0.01 μM). (**D**) Induction of FYN protein expression in SYO-1 cells after 4-8 h post FK228 treatment (0.01 μM). GAPDH serves as the loading control for western blot. Data represent mean ± SD of three independent experiments. ***P* < 0.01; ****P* < 0.001; *****P* < 0.0001; ns, not significant (two-tailed Student's *t*-test).

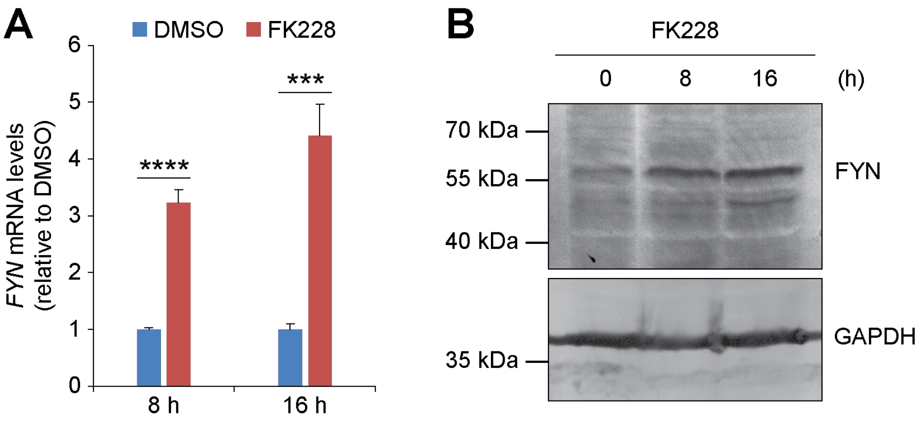

Figure S10. Effect of FK228 treatment on FYN expression in Yamato-SS cells.

(**A**) Induction of FYN mRNA expression in Yamato-SS cells treated with FK228 (0.1 μM) for 8-16 h. (**B**) Induction of FYN protein expression in Yamato-SS cells treated with FK228 (0.1 μM) for 8-16 h. GAPDH serves as the loading control. Data represent mean ± SD of three independent experiments (***$P < 0.001$; ****$P < 0.0001$, determined by two-tailed Student's *t*-test).

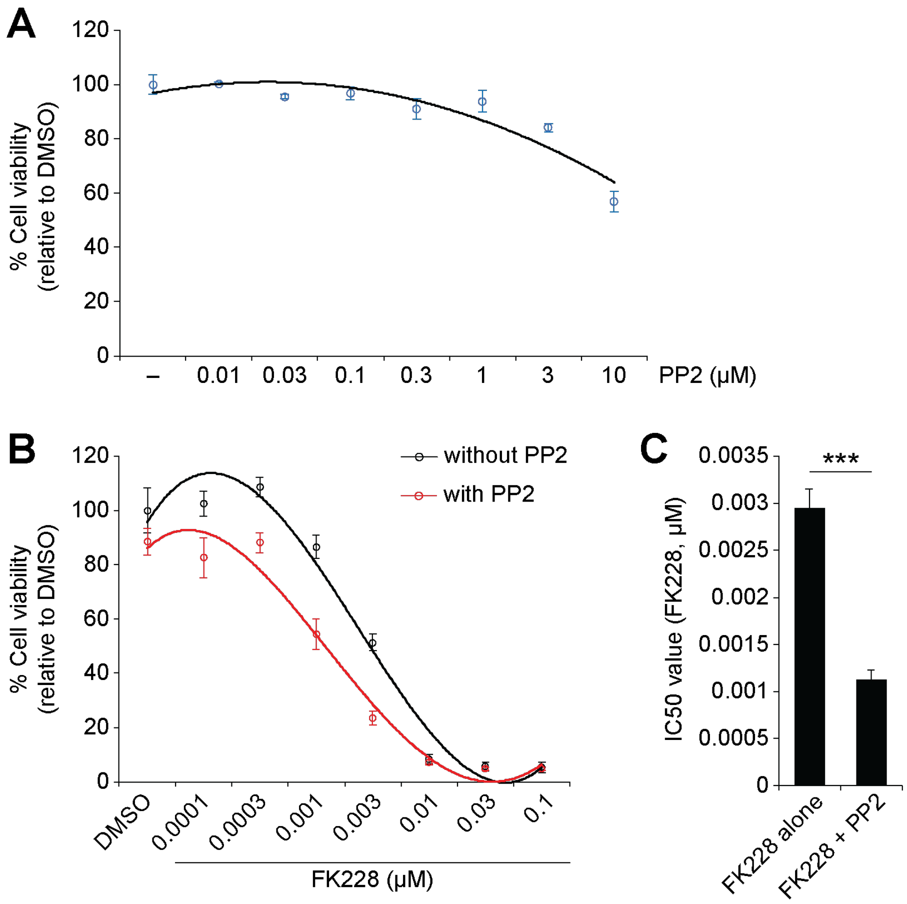

Figure S11. Effect of FK228 and PP2 treatment on the viability of SYO-1 cells.

(**A**) MTT-based viability analysis of SYO-1 cells treated with PP2 alone. (**B**) MTT-based viability analysis of SYO-1 cells treated with FK228 alone or in combination with PP2 (3 µM). (**C**) Comparison of IC50 values in SYO-1 cells treated with FK228 alone or in combination with PP2 (3 µM). Data represent mean ± SD of three independent experiments; ****P* < 0.001, determined by two-tailed Student's *t*-test.

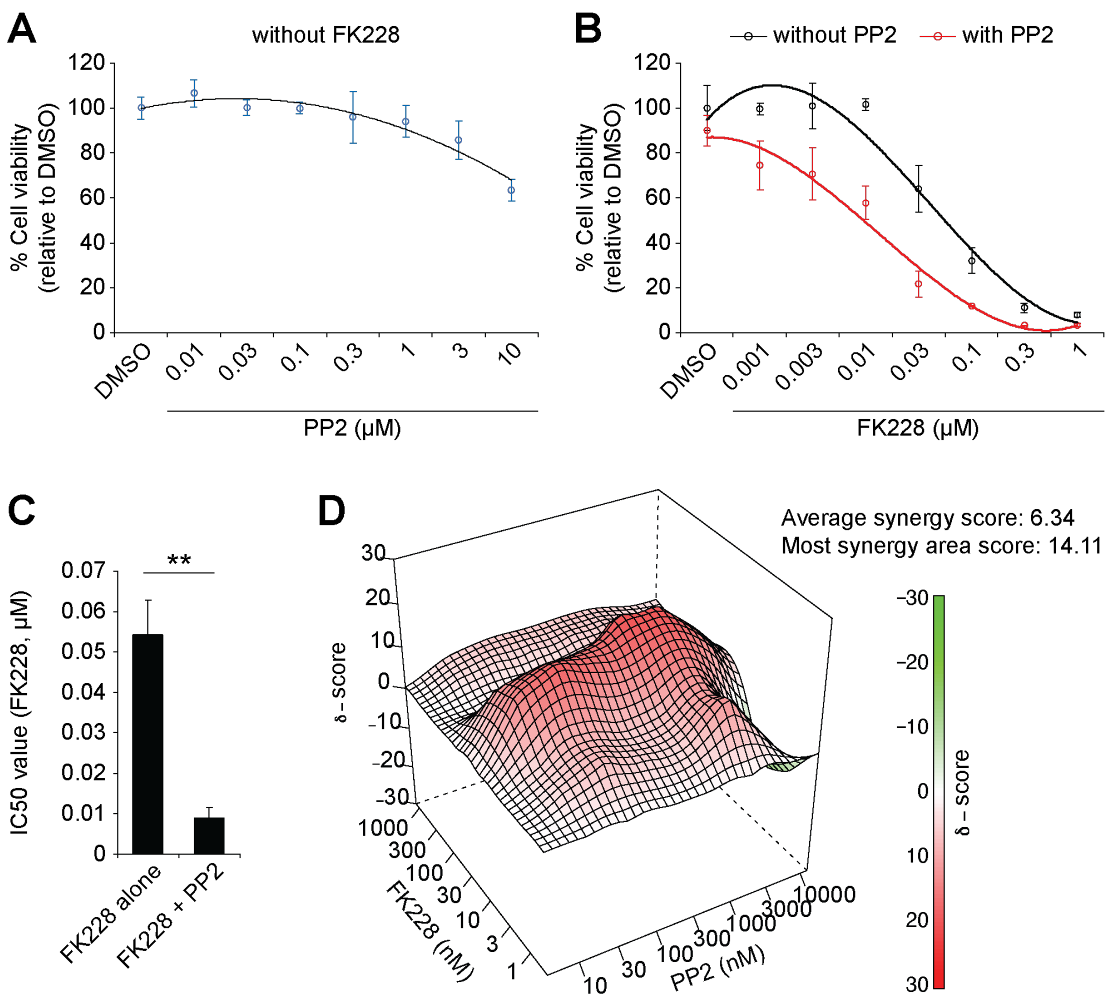

Figure S12. FK228 and PP2 display a synergistic effect in Yamato-SS cells.

(**A**) MTT-based viability analysis of Yamato-SS cells treated with PP2 alone. (**B**) MTT-based viability analysis of Yamato-SS cells treated with FK228 alone or in combination with PP2 (3 μM). (**C**) Comparison of IC50 values in Yamato-SS cells treated with FK228 alone or in combination with PP2 (3 μM). (**D**) 3D plot depicting a synergistic response between FK228 and PP2 in Yamato-SS cells. Data represent mean ± SD of three independent experiments; $^{**}P < 0.01$, determined by two-tailed Student's *t*-test.

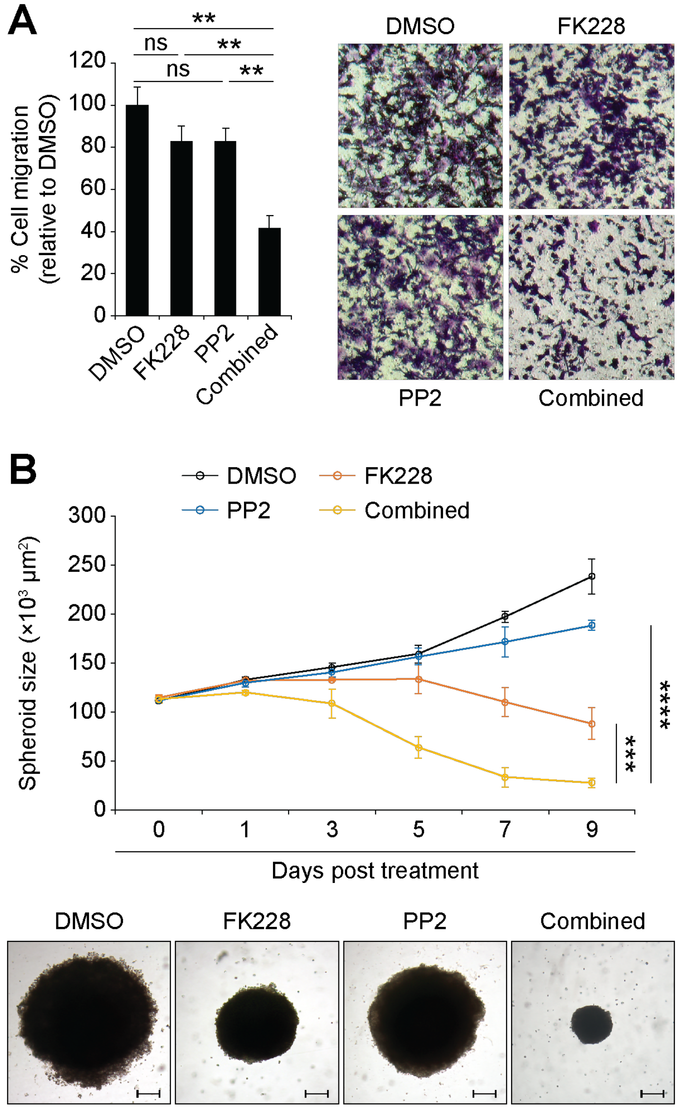

Figure S13. Effect of FK228 and PP2 treatment on Yamato-SS cell migration and 3D spheroid growth.

(**A**) Changes in the migration of Yamato-SS cells following exposure to either FK228 (0.1 μM) or PP2 (3 μM) alone or their combination. Migrated cells were stained with crystal violet (20× magnification). (**B**) Effects of FK228 and PP2 treatment alone or in combination on Yamato-SS spheroid growth. Representative images of Yamato-SS spheroids at day 7 in 3D cultures (scale bars, 100 μm). Data represent mean ± SD of 3-4 independent experiments. $^{**}P < 0.01$; $^{***}P < 0.001$; $^{****}P < 0.0001$; ns, not significant (two-tailed Student's *t*-test).

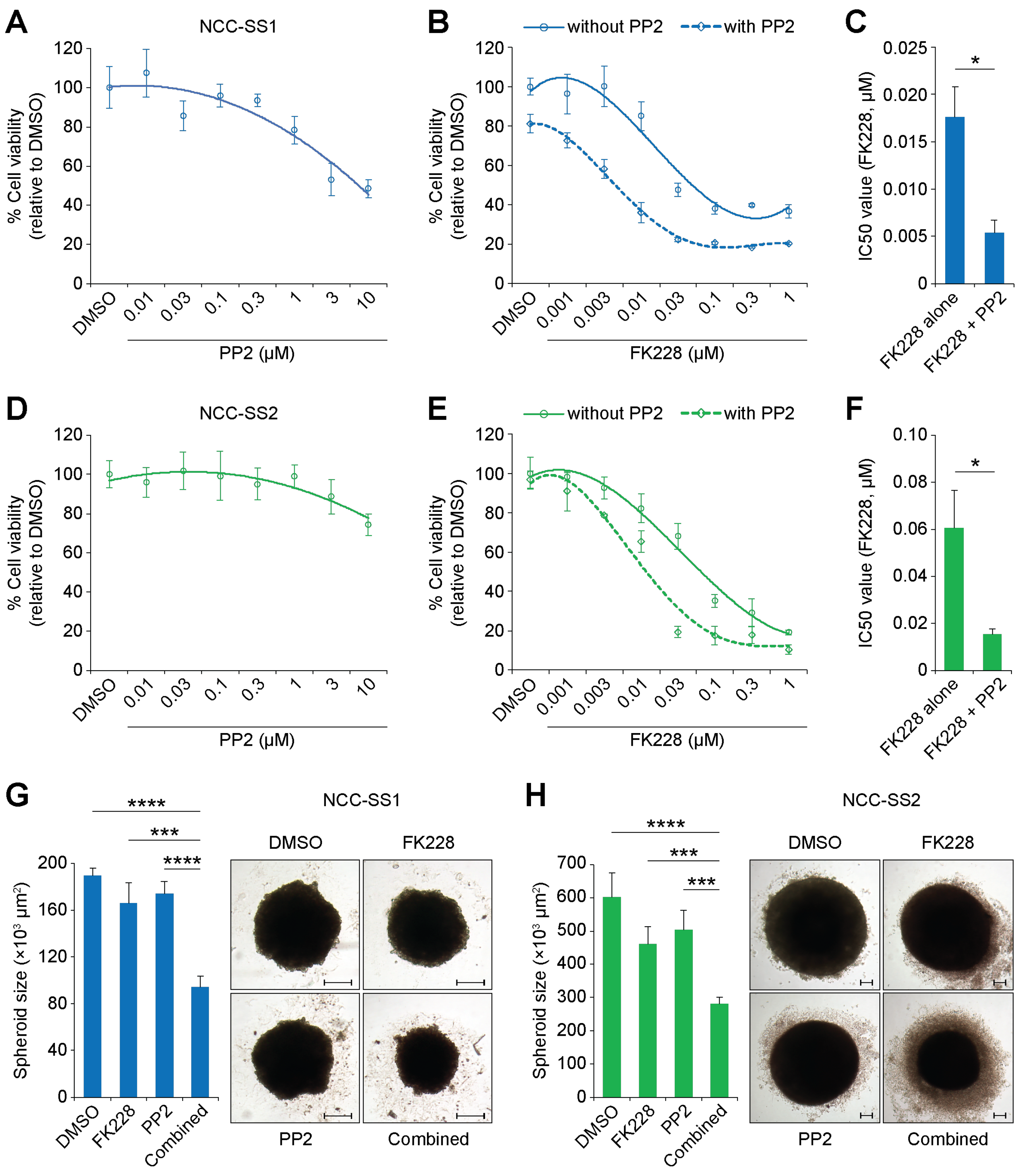

Figure S14. Effect of FK228 and PP2 on patient-derived synovial sarcoma cell growth in 2D and 3D culture.

(**A**) MTT-based viability analysis of NCC-SS1 cells treated with PP2 alone. (**B**) MTT-based viability analysis of NCC-SS1 cells treated with FK228 alone or in combination with PP2 (1 μM). (**C**) Comparison of IC50 values in NCC-SS1 cells treated with FK228 alone or in combination with PP2 (1 μM). (**D**) MTT-based viability analysis of NCC-SS2 cells treated with PP2 alone. (**E**) MTT-based

viability analysis of NCC-SS2 cells treated with FK228 alone or in combination with PP2 (1 μM). (**F**) Comparison of IC50 values in NCC-SS2 cells treated with FK228 alone or in combination with PP2 (1 μM). (**G** and **H**) Effect of FK228 and PP2 treatment alone or in combination on NCC-SS1 (**G**) and NCC-SS2 (**H**) spheroid growth. Representative images of multicellular spheroids at day 9 in 3D culture (scale bars, 100 μm). Data represent mean ± SD of 3-4 independent experiments. *$P < 0.05$; ***$P < 0.001$; ****$P < 0.0001$; ns, not significant (two-tailed Student's *t*-test).
